# The Three-Component Synthesis of 4-Sulfonyl-1,2,3-triazoles via a Sequential Aerobic Copper-Catalyzed Sulfonylation and Dimroth Cyclization

**DOI:** 10.3390/molecules26030581

**Published:** 2021-01-22

**Authors:** Max Van Hoof, Santhini Pulikkal Veettil, Wim Dehaen

**Affiliations:** Molecular Design and Synthesis, Department of Chemistry, KU Leuven, 3001 Leuven, Belgium; max.vanhoof@kuleuven.be (M.V.H.); santhini.pulikkalveettil@kuleuven.be (S.P.V.)

**Keywords:** 4-sulfonyl-1,2,3-triazoles, three-component tandem reaction, aerobic oxidation, copper-catalysis, C-H activation, Dimroth azide–enolate cyclization

## Abstract

4-Sulfonyl-1,2,3-triazole scaffolds possess promising bioactivities and applications as anion binders. However, these structures remain relatively unexplored and efficient synthetic procedures for their synthesis remain desirable. A practical room-temperature, aerobic copper-catalyzed three-component reaction of aromatic ketones, sodium sulfinates, and azides is reported. This procedure allows for facile access to 4-sulfonyl-1,5-disubstituted-1,2,3-triazoles in yields ranging from 34 to 89%. The reaction proceeds via a sequential aerobic copper(II)chloride-catalyzed oxidative sulfonylation and the Dimroth azide–enolate cycloaddition.

## 1. Introduction

**Figure 1:**
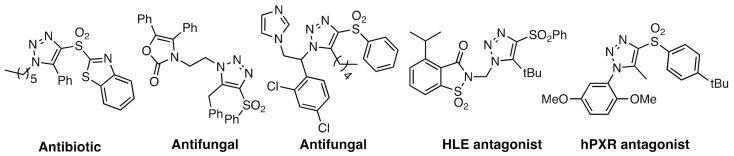
Examples of bioactive 4-sulfonyl triazoles.

4-Sulfonyl-1,2,3-triazole scaffolds possess very promising bioactivities, which include antibacterial [1] and antifungal agents [2,3], as well as potent antagonists of neutrophil elastase (HLE) [4] and the human pregnane X receptor (hPXR) [5,6,7], as shown in Figure 1. Additionally, they have found application as (monomeric) anion binders [8]. However, despite their promising applications, these structures remain relatively unexplored, making efficient synthetic procedures towards these scaffolds starting from readily available starting materials desirable.

**Scheme 1:**
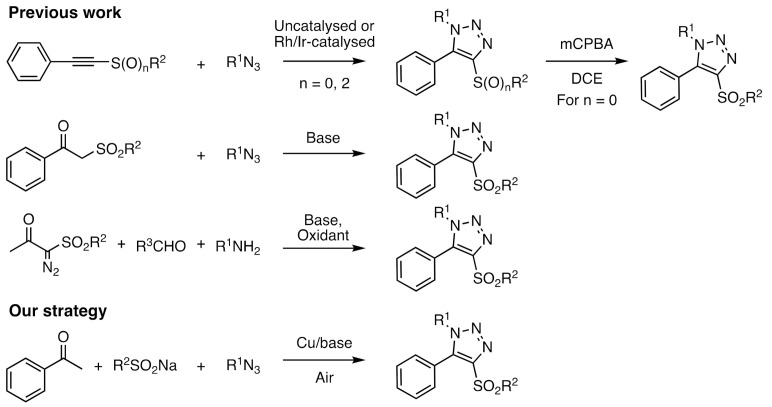
Synthetic routes towards 4-sulfonyl-triazoles.

Several methods for preparing 4-sulfonyl triazoles have been reported, each with their own drawbacks, as shown in Scheme 1. These routes include the non-catalyzed Huisgen azide–alkyne cycloadditions (AACs) of thio- or sulfonyl alkynes, which require high temperatures, long reaction times, and suffer from low yields and regioselectivity [4,9,10]. The metal catalyzed variants, of which the most notable are the IrAAC [11], RuAAC [12], and RhAAC [13], elegantly solve these issues. However, several limiting factors remain, such as the necessary multistep synthesis of the alkynyl sulfide [14,15,16,17] and sulfone starting materials [9,18,19,20], the use of stoichiometric oxidants for conversion of the thioether into a sulfone, and the high cost of the noble metal catalyst. The most prominent alternative to the AAC synthesis of 4-sulfonyl triazoles is the Dimroth azide–enolate cycloaddition of sulfonyl ketones [3,21,22,23,24]. While previously reported room temperature Dimroth cyclizations furnished 4-sulfonyl triazoles in good to high yields [3,22,23], the requirement of prior synthesis and isolation of the sulfonyl ketones reduces their sustainability and attractiveness [3,25,26,27,28,29,30,31,32,33,34,35]. Additionally, there are two alternative pathways, based on the Wolff triazole synthesis [36,37] and azide–alkene cycloaddition [38,39,40] that are both less attractive due to the use of unstable α-diazo-sulfonyl ketones, and the somewhat lower yields or availability of the starting materials. 

In light of the above-mentioned limitations, we envisioned a multicomponent 4-sulfonyl-1,2,3-triazole synthesis that starts from readily available aromatic ketones, sodium sulfinates, and organic azides, via a one-pot oxidative sulfonylation and Dimroth cyclization sequence, as shown in Scheme 1. In order to increase the sustainability of the envisioned procedure, we wanted to avoid the use of stoichiometric oxidants such as hypervalent iodine [25], peroxides [41], copper salts [42,43], and silver salts [44,45] that are commonly used in oxidative coupling reactions, in favor of air as the terminal oxidant, which is environmentally benign and results only in formation of water as a side product [43,46,47]. Over the past decade, many catalysts have been developed that allow for aerobic/O_2_ oxidative coupling of diverse substrates, with the significant focus being on palladium [48,49,50,51] and other noble metal catalysts [43,50,52,53,54,55]. However, copper catalysts are particularly interesting when compared to noble metal catalysts. Next to generally being inexpensive, readily available, and of low toxicity, they have great potential for catalyzing a broad range of reactions. Catalysis by natural copper-oxidases can serve as an example [46]. This is epitomized by the wide array of aerobic copper-catalyzed oxidative coupling reactions that have been reported to date [46,56,57].

## 2. Results and Discussion

As starting point for our three-component aerobic oxidative sulfonylation/Dimroth sequence, we utilized the reaction conditions reported by Lan et al. for the CuBr_2_-catalysed synthesis of α-alkyl ketosulfones, as shown in Table 1 and Table S1 [26]. While the original ligand-free procedure furnished the ketosulfones from α-unsubstituted acetophenone only in a low yield of 20%, it was expected that subsequent transformation of the in situ formed ketosulfone into the corresponding 1,2,3-triazole would result in a significantly improved yield. Pleasingly, when applying these reaction conditions, the corresponding 4-tosyl triazole **4a** was obtained in 54% yield (Entry 1). Different copper salts, including CuI, Cu(OAc)_2_, Cu(OTf)_2_, and CuCl_2_ were screened (Entries 1–5), and CuCl_2_ was found to be superior, furnishing **4a** in an isolated yield of 71%, and 72% upon repetition of the experiment (Entry 2). Next, different bases and solvents were evaluated, as well as a reduction in the equivalents of base (Entries 6–15, and Tables S16–S18). However, any deviation from the standard conditions (Entry 2) resulted in a decreased yield. The amine base may perform a dual role, acting both as base and ligand. Pyridine and Et_3_N (Entry 6 and 7) presumably are unreactive because they are both weaker bases and ligands than DBU, thereby not sufficiently deprotonating the enol and stabilizing the copper complex. Surprisingly, DBN results in a markedly lower yield than DBU (Entry 8), 52% versus 72%, which may be the result of it being too strongly coordinated to the copper and thereby hindering the formation of the copper-enolate. K_2_CO_3_ and KOtBu presumably are unreactive since they are both less soluble in DMSO and weaker ligands (Entry 9 and 10). Presumably, DMSO is superior since it is both a polar coordinating solvent as well as a mild oxidant. 

Subsequently, different tertiary amine ligands were screened, including TMEDA, 2,2′-bipyridine, 1,10-phenanthroline, and neocuproine (Entries 16–19). Primary and secondary amine ligands were excluded due to the risk of α-amination [58], and phosphine ligands were excluded due to their susceptibility to oxidative degradation [51]. Out of the evaluated amine ligands, TMEDA proved to be the superior with a yield of 81% (Entry 16). Finally, the catalyst loading and solvent volume were varied and an optimal loading of 10 mol% CuCl_2_/TMEDA and 2 mL volume of DMSO was found, resulting in a yield of 89% (Entry 28). As final control experiments, reactions were set up under the optimized conditions in either absence of CuCl_2_ (Entry 29) or under argon atmosphere (Entry 30), which resulted in no reaction and in less than 5% of triazole being formed.

**Scheme 2:**
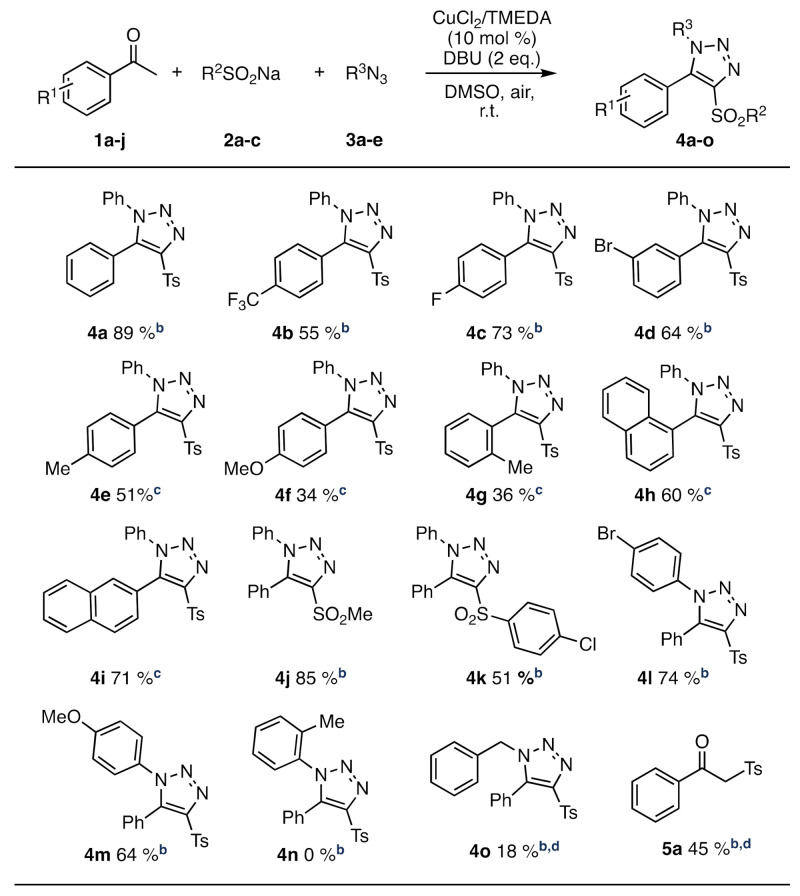
Substrate scope for three-component 4-sulfonyl triazole synthesis**. ^a^** Reaction conditions: Acetophenone **1** (0.50 mmol), sodium sulfinate **2** (1.00 mmol), DBU (1.00 mmol), CuCl_2_/TMEDA (10%) in DMSO (2 mL) under air atmosphere. ^b^ 24 h. ^c^ 48 h. ^d^ 35 °C. Ts = p-toluene sulfonyl.

With the optimized conditions in hand, we set out to explore the substrate scope for this reaction and investigated various acetophenone derivatives, sodium sulfinates, and organic azides, as shown in Scheme 2. For the reaction of acetophenone derivatives **1a**–**j** with sodium p-toluene sulfinate **2a** and phenyl azide **3a**, the yields of triazoles **4a**–**j** varied from high to low. The reactions of electron-deficient acetophenones progressed at similar or faster rates yet resulted in lower yields. The 4-tosyl triazoles derived from p-trifluoromethyl substituted **4b**, p-fluoro substituted **4c**, and o-bromo substituted **4d** acetophenone were obtained in yields of 55 (**4b**), 73 (**4c**), and 64% (**4d**). The reduced yields compared to **4a** can be in part explained by the occurrence of Regitz diazo transfer, as observed previously for cyclic sulfonyl ketones [24] and evidenced by the observation of nitrogen evolution from the reaction mixture and the presence of a minor quantity of aniline in the crude mixture, as determined by GC/MS. However, no other product from this side-reaction could be isolated. The presence of donating substituents resulted in a reduced reaction rate and concomitantly reduced yields. The p-methyl and p-methoxy substituted triazoles **4e** and **4f** were obtained in yields of 51% and 34% after 48 h. Next, the influence of steric hindrance was investigated and the expected negative correlation between steric hindrance and product yields was observed. The o-methyl and 1-naphthyl triazoles **4g** and **4h** give reduced yields in comparison to their less sterically hindered counterparts, the p-methyl and 2-naphthyl triazoles **4e** and **4i**, 36% (**4g**) versus 51% (**4e**) and 60% (**4h**) versus 71% (**4i**). Several aliphatic ketones including acetone, pinacolone, and trifluoromethyl acetone were screened without success, as expected.

A plausible reason for the limitations of the reaction scope for ketones is given in the final paragraph, after discussing the reaction mechanism.

Pleasingly, 4-methyl sulfinate triazole **4j** was furnished in a good yield of 85%. However, in the case of the p-chlorophenyl sulfonyl triazole **4k**, a yield of only 51% was obtained. Finally, the scope in azide was evaluated and both for phenyl azides with electron-withdrawing and donating groups, good yields of 74% (**4l**) and 64% (**4m**) were obtained. Regrettably, sterically hindered azides were unreactive and no triazole **4n** formed. Benzyl azide performed far less effectively and furnished the triazole **4o** in 18% yield, along with 45% of sulfonyl ketone **5a**. 

A plausible reason for the limitations of the reaction scope for azides is given in the final paragraph, after discussing the reaction mechanism.

**Scheme 3:**
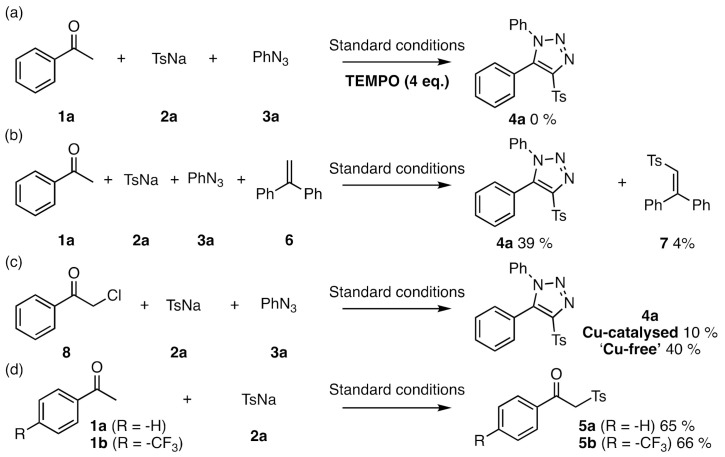
Mechanistic control experiments. (**a**) Reaction in presence of TEMPO. (**b**) Reaction in presence of DPE **6**. (**c**) Reaction of phenacyl chloride under standard and copper-free conditions. (**d**) Reaction in absence of azide **3a**.

In order to gain more insight into the mechanism, several control experiments were performed. In the absence of CuCl_2_ no reaction occurred, and under argon atmosphere less than 5% of product formed, as shown in Table S1 (Entry 32–33), which shows that the copper salt is required for catalyzing the reaction and oxygen is needed for catalytic turnover. Addition of four equivalents of TEMPO completely inhibited the reaction, as shown in Scheme 3a. From the control reaction in the presence of 1,1-diphenylethylene (DPE) **6**, a radical trapping reagent, the sulfonyltriazole **4a**, could be isolated in a reduced yield of 39% and the yield of the radical trapping product **7** was rather low, 4%, as shown in Scheme 3b. These results indicate involvement of both free radicals and alternative mechanisms, such as via oxidative chlorination or via an organometallic intermediate. 

**Scheme 4:**
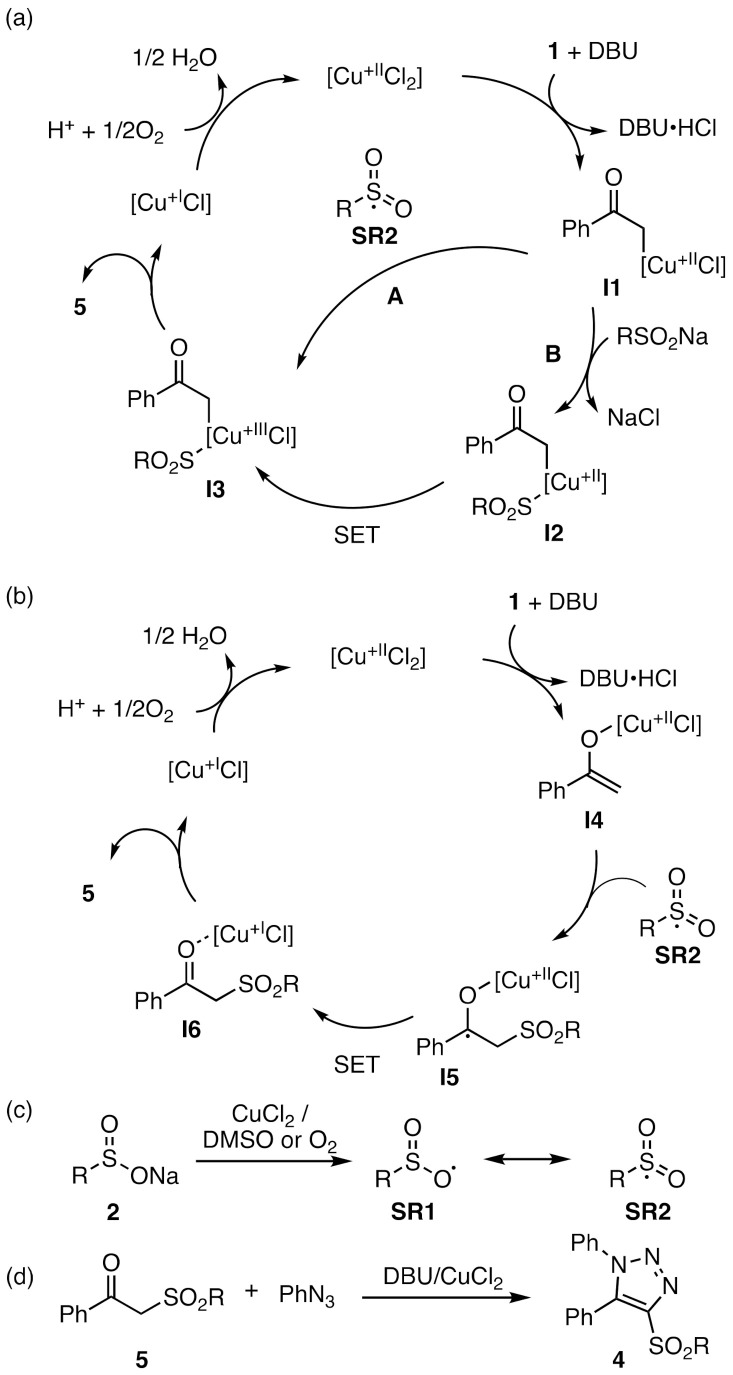
Proposed mechanism for three-component reaction for (**a**) oxidative sulfonylation via Cu(III)-intermediate, (**b**) oxidative sulfonylation via Cu(II)-intermediate, (**c**) generation of sulfonyl radical, and (**d**) enolate/azide cycloaddition.

Phenacyl chloride **8** is a possible reaction intermediate, considering that the halogenation of the carbonyl α-position by stoichiometric copper halides, as well as diverse aerobic copper-catalyzed oxidative halogenations, have been reported [46,59,60]. From the reaction of **8** under standard conditions in presence and absence of the copper catalyst, triazole **4a** was obtained in yields of 11% and 45%, as shown in Scheme 4c. This indicates that **8** may be an intermediate in the oxidative sulfonylation, although, it shows that there must be alternative operative pathways. However, it should be noted that **8** was not isolated from or observed in the reaction mixture. Additionally, the reported copper-catalyzed oxidative halogenation reactions generally take place under (Lewis) acidic conditions, which raise the Cu(II) reduction potential and consequently promote single-electron transfer SET reactivity. Conversely, basic conditions and stronger ligands stabilize both Cu(II) and Cu(III), or lower the Cu(II) reduction potential, which reduces SET reactivity and favors the formation of organometallic intermediates [46,61]. 

The intermediacy of the sulfonyl ketone **5** is supported by the observation of the NMR characteristic peak in the crude reaction mixture at reduced reaction times. When the oxidative sulfonylation was performed in absence of azide good yields of phenacyl sulfones **5a** and **5b** were obtained, as shown in Scheme 3d, which proves its intermediacy. Advantageously, this shows that the presented methodology can also be used for the synthesis of sulfonyl ketones in good yield.

Based on the reaction outcomes and literature reports [26,27,46,62,63,64,65,66,67], three plausible mechanisms are considered, as shown in Scheme 4, two of which are analogous to those described by Lan et al. (Scheme 4a pathway A and Scheme 4b) [26]. First, DBU deprotonates the acetophenone **1**, and the resulting enolate exchanges chloride at Cu(II), forming intermediate **I1**. Next, there are two possible pathways, as shown in Scheme 4a. Via pathway A, the free sulfonyl radical **SR2** is formed through oxidation of the sulfinate **2** by Cu(II), oxygen, or DMSO via a SET mechanism [63,64,65,66,67], as shown in Scheme 4b, and then undergoes oxidative addition to the Cu(II) complex, forming Cu(III)-intermediate **I3** [26,27,66]. Via pathway B, ligand exchange of the chloride for sulfinate results in the formation of Cu(II)-intermediate **I2**. This intermediate can then be oxidized via SET by CuCl_2_, or by oxygen or DMSO, resulting in the formation of Cu(III)-intermediate **I3.** Reductive elimination of **I3** results in the formation of sulfonyl ketone **5** and Cu(I)Cl. Finally, oxidation of Cu(I) by oxygen or DMSO regenerates the Cu(II)-catalyst [26,27,46,62,66]. 

In the third pathway, as shown in Scheme 4b, the sulfonyl radical **SR2** reacts with copper enolate **I4** with formation of a benzylic ketyl radical **I5**. Oxidation of the ketyl radical **I5** by Cu(II) via an intramolecular SET process forms the ketosulfone **5** and Cu(I)Cl, which is reoxidized by oxygen to regenerate the active copper(II) catalyst [46,62,63,64,65,66,67]. The sulfonyl ketone **5** can undergo DBU Bronsted-basic and/or CuCl_2_ Lewis-acidic mediated Dimroth enolate/azide cycloaddition, forming the 4-sulfonyl triazole **4**. 

The reason why the reaction scope is limited to aromatic ketones can be explained by the reaction mechanisms shown in Scheme 4, in which the first step involves the formation of a (copper-) enolate. The observation that the reaction times increase for more electron-rich aromatic ketones indicates that this deprotonation may be the rate limiting step. This fact would explain why acetone and pinacolone are unreactive since these are less acidic than acetophenone by at least 1.8 orders of magnitude. On the other hand, 1,1,1,-trifluoroacetone, while more acidic, may be unreactive due to its high tendency to form hydrates with water [68]. The reason for the reduced yields of 1,2,3-triazole **4o** from the reaction with benzyl azide are intrinsic to the Dimroth azide–enolate cycloaddition, which was defined by L’abbé in 1971 as “the condensation of organic azides with active methylene compounds in the presence of an equimolar amount of organic or inorganic base leading to highly substituted 1,2,3-triazoles in a regioselective manner” [69,70]. The reaction mechanism involves either a stepwise concerted [3+2] cycloaddition or a stepwise addition of the enolate to the azide with formation of an N1-triazenyl ion intermediate. The rate of this cycloaddition depends on the stabilization of this ion [71]. For this reason, aromatic azides with electron-donating groups are expected to react more slowly than those with withdrawing substituents, and benzyl azides and aliphatic azides are expected to react more slowly than their aromatic counterparts.

## 3. Materials and Methods

### 3.1. General Information 

All chemicals were purchased from Acros Organics (Geel, Belgium), Merck (Darmstadt, Germany), Alfa Aesar (Kandel, Germany), Fluorochem (Hadfield, UK), and TCI Europe (Zwijndrecht, Belgium) and used as received. For column chromatography, 70–230 mesh silica 60 (Acros, Geel, Belgium) was used as the stationary phase. NMR data were recorded using a Bruker AV 400 MHz (Bruker (Biospin), Kontich, Belgium) and chemical shifts (δ) were reported in parts per million (ppm) referenced to tetramethylsilane (^1^H), or the internal (NMR) solvent signal (^13^C) as internal standards [72]. High-resolution mass spectra were acquired on a quadrupole orthogonal acceleration time-of-flight mass spectrometer (Synapt G2 HDMS, Waters, Milford, MA, USA). Samples were infused at 3 μL/min and spectra were obtained in positive ionization mode with a resolution of 15,000 (FWHM—full width at half maximum) using leucine enkephalin as a lock mass. Melting points (not corrected) were determined using a Reichert Thermovar apparatus. GC/MS were measured on a Thermo Finnigan Interscience Trace™ GC gas chromatographer (Waltham, Massachusetts, USA) coupled to a Thermo Scientific ITQ 900™ mass spectrometer (Waltham, Massachusetts, USA) in full-scan EI (electron ionization) mode. Phenyl azide (**3a**), 4-bromophenyl azide (**3b**), 4-methoxyphenyl azide (**3c**), and benzyl azide (**3d**) were prepared according to literature procedures [40,73].

FAIR Data is available as Supporting Information for Publication and includes the primary NMR FID files for compounds: **4a**, **4b**, **4c**, **4d**, **4e**, **4f**, **4g**, **4h**, **4i**, **4j**, **4k**, **4l**, **4m**, **4o**, **5a**, **5b**.

### 3.2. General Procedure and Characterization Data

To a 10 mL round bottom flask, acetophenone **1a**–**j** (0.50 mmol), DMSO (dry, 2 mL), sodium sulfinate **2a**–**c** (1.00 mmol, 2 eq.), DBU (150 µL, 1.00 mmol, 2 eq.), TMEDA (8 µL, 0.05 mmol, 10 mol%), and azide **3a**–**e** (0.75 mmol, 1.5 eq.) were added sequentially. The reaction was initiated by addition of CuCl_2_ (6.7 mg, 10 mol%). After stirring open vessel for 24 or 48 h at 25 °C, 5 mL EtOAc and 3 mL NH_3_Cl were added and the mixture was transferred to a separation funnel along with 20 mL EtOAc. Then, 3 mL H_2_O was added in order to break the suspension. The organic phase was collected and following two more extractions (2 × 25 mL EtOAc), washed with brine, dried over Na_2_SO_4_, and concentrated in vacuo. Flash chromatography with ethyl acetate/petroleum ether 10–60% as eluent afforded the title products as off-white and white solids.

*1,5-Diphenyl-4-[(4-methylphenyl)sulfonyl]-1H-1,2,3-triazole* (**4a**), Prepared according to the general procedure using acetophenone **1a** (60 mg, 0.50 mmol), sodium p-toluenesulfinate **2a** (178 mg, 1.00 mmol), and phenyl azide **3a** (83 μL, 0.75 mmol). Reaction time was 24 h. Flash chromatography using EtOAc/petroleum ether 10–40% yielded **4a** (170 mg, 0.453 mmol, 89%) as a white solid; mp 175–177 °C. ^1^H-NMR (400 MHz, CDCl_3_): δ (ppm) 7.82–7.71 (app. d, *J* = 8.3 Hz, 2H), 7.50–7.43 (m, 1H), 7.43–7.30 (m, 5H), 7.30–7.17 (m, 6H), 2.39 (s, 3H). ^13^C-NMR (101 MHz, CDCl_3_): δ (ppm) 146.1, 144.9, 138.6, 137.7, 135.4, 130.5, 130.4, 129.9, 129.8, 129.4, 128.6, 128.1, 125.1, 124.3, 21.7. HRMS (ESI-Q-TOF): *m*/*z* [M + H]^+^ calcd. for C_21_H_17_N_3_O_2_S_1_: 376.11141; found: 376.1113.

*1-Phenyl-4-[(4-methylphenyl)sulfonyl]-5-(4-trifluoromethylphenyl)-1H-1,2,3-triazole* (**4b**), Prepared according to the general procedure using 4′-(trifluoromethyl)acetophenone **1b** (94 mg, 0.50 mmol), sodium p-toluenesulfinate **2a** (178 mg, 1.00 mmol), and phenyl azide **3a** (83 μL, 0.75 mmol). Reaction time was 24 h. Flash chromatography using EtOAc/petroleum ether 10–40% yielded **4b** (122 mg, 0.275 mmol, 55%) as an off-white solid; mp 208–210 °C. ^1^H-NMR (400 MHz, CDCl_3_): δ (ppm) 7.86–7.76 (app. d, *J* = 8.1 Hz, 2H), 7.71–7.60 (app. d, *J* = 8.1 Hz, 2H), 7.50–7.34 (m, 5H), 7.33–7.27 (app. d, *J* = 8.0 Hz, 2H), 7.24–7.16 (app. d, *J* = 7.7, 2H), 2.42 (s, 3H). ^13^C-NMR (101 MHz, CDCl_3_): δ (ppm) 146.8, 145.4, 137.4, 137.1, 135.2, 132.5 (q, *J* = 33.1 Hz), 131.2, 130.3, 130.0, 129.8, 128.4, 125.7 (q, *J* = 3.6 Hz), 125.3, 123.6 (q, *J* = 272.7 Hz), 21.8. ^19^F-NMR (377 MHz, CDCl_3_): δ (ppm)–63.00 Hz. HRMS (ESI-Q-TOF): *m*/*z* [M + H]^+^ calcd. for C_22_H_16_F_3_N_3_O_2_S_1_: 444.09879; found: 444.0987. 

*1-Phenyl-4-[(4-methylphenyl)sulfonyl]-5-(4-fluorophenyl)-1H-1,2,3-triazole* (**4c**), Prepared according to the general procedure using 4′-fluoroacetophenone **1c** (68 mg, 0.49 mmol), sodium p-toluenesulfinate **2a** (178 mg, 1.00 mmol), and phenyl azide **3a** (83 μL, 0.75 mmol). Reaction time was 24 h. Flash chromatography using EtOAc/petroleum ether 10–40% yielded **4c** (122 mg, 0.359 mmol, 73%) as an off-white solid; mp 150–152 °C. ^1^H-NMR (400 MHz, CDCl_3_): δ (ppm) 7.83–7.70 (app. d, *J* = 8.2 Hz, 2 H), 7.47–7.33 (m, 3 H), 7.32–7.24 (m, 4H), 7.33–7.25 (app. d, *J* = 7.1 Hz, 2H), 7.13–7.03 (app. t, *J* = 8.6 Hz, 2 H), 2.40 (s, 3H). ^13^C-NMR (101 MHz, CDCl_3_): δ (ppm) 163.9 (d, *J* = 252.3 Hz) 146.3, 145.1, 137.7, 137.6, 135.3, 132.7 (d, *J* = 8.8 Hz), 130.1, 129.9, 129.6, 128.2, 125.3, 120.4 (d, *J* = 3.5 Hz), 116.0 (d, *J* = 22.2 Hz), 21.7. ^19^F-NMR (377 MHz, CDCl_3_): δ (ppm)—108.69 Hz. HRMS (ESI-Q-TOF): *m*/*z* [M + H]^+^ calcd. for C_21_H_16_F_1_N_3_O_2_S_1_: 394.10199; found: 394.1018.

*1-Phenyl-4-[(4-methylphenyl)sulfonyl]-5-(3-bromomethylphenyl)-1H-1,2,3-triazole* (**4d**), Prepared according to the general procedure using 4′-bromoacetophenone **1d** (100 mg, 0.500 mmol), sodium p-toluenesulfinate **2a** (178 mg, 1.00 mmol), and phenyl azide **3a** (83 μL, 0.75 mmol). Reaction time was 24 h. Flash chromatography using EtOAc/petroleum ether 10–40% yielded **4d** (145 mg, 0.319 mmol, 64%) as an off-white solid; mp 158–160 °C. ^1^H-NMR (400 MHz, CDCl_3_): δ (ppm) 7.85–7.75 (app. d, *J* = 8.2 Hz, 2H), 7.63–7.55 (app. d, *J* = 7.9 Hz, 1H), 7.47–7.34 (m, 4H), 7.33–7.18 (m, 6, H), 2.42 (s, 3H). ^13^C-NMR (101 MHz, CDCl_3_): δ (ppm) 146.6, 145.3, 137.5, 137.0, 135.2, 133.7, 133.2, 130.22, 130.20, 130.0, 129.7, 129.2, 128.3, 126.4, 125.2, 122.5, 21.8. HRMS (ESI-Q-TOF): *m*/*z* [M + H]^+^ calcd. for C_21_H_16_Br_1_N_3_O_2_S_1_: 454.02198; found: 454.0216. 

*1-Phenyl-4-[(4-methylphenyl)sulfonyl]-5-(4-methylphenyl)-1H-1,2,3-triazole* (**4e**), Prepared according to the general procedure using 4′-methylacetophenone **1e** (67 mg, 0.49 mmol), sodium p-toluenesulfinate **2a** (178 mg, 1.00 mmol), and phenyl azide **3a** (83 μL, 0.75 mmol). Reaction time was 24 h. Flash chromatography using EtOAc/petroleum ether 10–40% yielded **4e** (97 mg, 0.25 mmol, 51%) as an off-white solid; mp 116–118 °C. ^1^H-NMR (400 MHz, CDCl_3_): δ (ppm) 7.83–7.73 (app. d, *J* = 8.3 Hz, 2H), 7.43–7.22 (m, 3 H), 7.30–7.24 (m, 2H), 7.24–7.11 (m, 6H), 2.40 (s, 3H), 2.38 (s, 3H). ^13^C-NMR (101 MHz, CDCl_3_): δ (ppm) 146.1, 144.9, 140.9, 138.9, 137.9, 135.7, 130.4, 129.8, 129.5, 129.4, 128.3, 125.3, 121.3, 21.7, 21.6. HRMS (ESI-Q-TOF): *m*/*z* [M + H]^+^ calcd. for C_22_H_19_N_3_O_2_S_1_: 390.12706; found: 390.1269.

*1-Phenyl-4-[(4-methylphenyl)sulfonyl]-5-(4-methoxyphenyl)-1H-1,2,3-triazole* (**4f**), Prepared according to the general procedure using 4′-methoxyacetophenone **1f** (75 mg, 0.50 mmol), sodium p-toluenesulfinate **2a** (178 mg, 1.00 mmol), and phenyl azide **3a** (83 μL, 0.75 mmol). Reaction time was 48 h. Flash chromatography using EtOAc/petroleum ether 10–60% yielded **4f** (70 mg, 0.17 mmol, 34%) as an off-white solid; mp 122–125 °C. ^1^H-NMR (400 MHz, CDCl_3_): δ (ppm) 7.82–7.73 (app. d, *J* = 8.3 Hz, 2H), 7.44–7.31 (m, 3 H), 7.30–7.16 (m, 6 H), 6.92–6.84 (app. d, *J* = 8.8 Hz, 2H), 3.83 (s, 3H), 2.39 (s, 3H). ^13^C-NMR (101 MHz, CDCl_3_): δ (ppm) 161.2, 145.8, 144.9, 138.7, 137.8, 135.6, 132.0, 129.8, 129.4, 128.1, 125.2, 116.0, 114.2, 55.4, 21.7. HRMS (ESI-Q-TOF): *m*/*z* [M + H]^+^ calcd. for C_22_H_19_N_3_O_3_S_1_: 406.12198; found: 406.1212. 

*1-Phenyl-4-[(4-methylphenyl)sulfonyl]-5-(4-methoxyphenyl)-1H-1,2,3-triazole* (**4g**), Prepared according to the general procedure using 2′-methyl acetophenone **1g** (67 mg, 0.50 mmol), sodium p-toluenesulfinate **2a** (178 mg, 1.00 mmol), and phenyl azide **3a** (83 μL, 0.75 mmol). Reaction time was 48 h. Flash chromatography using EtOAc/petroleum ether 10–40% yielded **4g** (69 mg, 0.18 mmol, 36%) as a white solid; mp 154–156 °C. ^1^H-NMR (400 MHz, CDCl_3_): δ (ppm) 7.75–7.65 (app. d, *J* = 8.3 Hz, 2 H), 7.44–7.13 (m, 11H), 2.40 (s, 3H), 1.73 (s, 3H). ^13^C-NMR (101 MHz, CDCl_3_): δ (ppm) 146.6, 144.9, 138.5, 137.79, 137.77, 135.8, 131.1, 131.0, 130.6, 129.8, 129.5, 128.2, 126.13, 124.3, 124.1, 21.8, 19.7. HRMS (ESI-Q-TOF): *m*/*z* [M + H]^+^ calcd. for C_22_H_19_N_3_O_2_S_1_: 390.12706; found: 390.1266.

*1-Phenyl-4-[(4-methylphenyl)sulfonyl]-5-(1-naphtyl)-1H-1,2,3-triazole* (**4h**), Prepared according to the general procedure using 1-acetonaphtone **1h** (84 mg, 0.49 mmol), sodium p-toluenesulfinate **2a** (178 mg, 1.00 mmol), and phenyl azide **3a** (83 μL, 0.75 mmol). Reaction time was 48 h. Flash chromatography using EtOAc/petroleum ether 10–40% yielded **4h** (126 mg, 0.296 mmol, 60%) as a white solid; mp = 195–197 °C. ^1^H-NMR (400 MHz, CDCl_3_): δ (ppm) 8.99–7.92 (app. d, *J* = 8.2 Hz, 1H), 7.89–7.80 (app. d, *J* = 8.2 Hz, 1H), 7.59–7.47 (m, 3H), 7.46–7.38 (m, 2H), 7.29–7.12 (m, 6H), 7.09–6.99 (app. d, *J* = 8.1 Hz, 2H), 7.98–7.88 (app. d, *J* = 8.4 Hz, 1H), 2.29 (s, 3H). ^13^C-NMR (101 MHz, CDCl_3_): δ (ppm) 147.7, 144.7, 137.5, 137.3, 135.7, 133.3, 131.6, 131.2, 130.0, 129.8, 129.5, 129.3, 128.7, 128.2, 127.3, 125.6, 125.0, 124.3, 124.2, 122.0, 21.6. HRMS (ESI-Q-TOF): *m*/*z* [M + H]^+^ calcd. for C_25_H_19_N_3_O_2_S_1_: 426.12706; found: 426.1269.

*1-Phenyl-4-[(4-methylphenyl)sulfonyl]-5-(2-naphtyl)-1H-1,2,3-triazole* (**4i**), Prepared according to the general procedure using 2-acetonaphtone **1i** (84 mg, 0.49 mmol), sodium p-toluenesulfinate **2a** (178 mg, 1.00 mmol), and phenyl azide **3a** (83 μL, 0.75 mmol). Reaction time was 24 h. Flash chromatography using EtOAc/petroleum ether 10–50% yielded **4i** (149 mg, 0.350 mmol, 71%) as a white solid; mp 206–208 °C. ^1^H-NMR (400 MHz, CDCl_3_): δ (ppm) 7.93–7.69 (m, 6 H), 7.64–7.47 (m, 2H), 7.43–7.09 (m, 8H), 2.36 (s, 3H). ^13^C-NMR (101 MHz, CDCl_3_): δ (ppm) 146.5, 144.9, 138.7, 137.8, 135.6, 133.8, 132.6, 131.2, 129.9, 129.8, 129.5, 128.6, 128.5, 128.3, 127.9, 127.9, 127.1, 126.4, 125.2, 121.6, 21.7. HRMS (ESI-Q-TOF): *m*/*z* [M + H]^+^ calcd. for C_25_H_19_N_3_O_2_S_1_: 426.12706; found: 426.1265.

*1,5-Diphenyl-4-methylsulfonyl-1H-1,2,3-triazole* (**4j**), Prepared according to the general procedure using acetophenone **1a** (60 mg, 0.50 mmol), sodium methanesulfinate **2b** (110 mg, 1.00 mmol), and phenyl azide **3a** (83 μL, 0.75 mmol). Reaction time was 24 h. Flash chromatography using EtOAc/petroleum ether 10–60% yielded **4j** (127 mg, 0.424 mmol, 85%) as a white solid; mp 155–157 °C. ^1^H-NMR (400 MHz, CDCl_3_): δ (ppm) 7.52–7.32 (m, 8H), 7.32–7.20 (app. d, *J* = 7.46 Hz, 2H), 2.34 (s, 3H). ^13^C-NMR (101 MHz, CDCl_3_): δ (ppm) 145.3, 138.4, 135.5, 130.8, 130.5, 130.1, 129.6, 128.8, 125.4, 123.9, 43.5. HRMS (ESI-Q-TOF): *m*/*z* [M + H]^+^ calcd. for C_15_H_13_N_3_O_3_S_1_: 300.08011; found: 300.0800. 

*1,5-Diphenyl-4-[(4-chlorophenyl)sulfonyl]-1H-1,2,3-triazole* (**4k**), Prepared according to the general procedure using acetophenone **1a** (60 mg, 0.50 mmol), sodium 4-chlorophenylsulfinate **2c** (198 mg, 1.00 mmol), and phenyl azide **3a** (83 μL, 0.75 mmol). Reaction time was 24 h. Flash chromatography using EtOAc/petroleum ether 10–60% yielded **4k** (100 mg, 0.253 mmol, 85%) as an off-white solid; mp = 149–151 °C. ^1^H-NMR (400 MHz, CDCl_3_): δ (ppm) 7.90–7.74 (app. d, *J* = 8.3 Hz), 7.63–7.32 (m, 8 H), 7.32–7.13 (m, 4H). ^13^C-NMR (101 MHz, CDCl_3_): δ (ppm) 145.6, 140.7, 139.2, 139.0, 135.4, 130.7, 130.5, 130.0, 129.7, 129.5, 128.8, 125.2, 124.2. HRMS (ESI-Q-TOF): *m*/*z* [M + H]^+^ calcd. for C_20_H_14_Cl_1_N_3_O_3_S_1_: 396.05679; found: 396.0558. 

*1-(4-Bromophenyl)-4-[(4-methylphenyl)sulfonyl]-5-phenyl-1H-1,2,3-triazole* (**4l**), Prepared according to the general procedure using acetophenone **1a** (60 mg, 0.50 mmol), sodium p-toluenesulfinate **2a** (178 mg, 1.00 mmol), and 4-bromophenyl azide **3b** (149 mg, 0.750 mmol). Reaction time was 24 h. Flash chromatography using EtOAc/petroleum ether 10–40% yielded **4l** (167 mg, 0.368 mmol, 74%) as a pale brown solid; mp 192–194 °C. ^1^H-NMR (400 MHz, CDCl_3_): δ (ppm) 7.82–7.73 (app. d, *J* = 8.3 Hz, 2H), 7.53–7.46 (m, 3H), 7.46–7.38 (m, 2H), 7.32–7.24 (m, 4H), 7.15–7.07 (app d, *J* = 8.8 Hz, 2H), 2.41 (s, 3H). ^13^C-NMR (101 MHz, CDCl_3_): δ (ppm) 146.5, 145.1, 138.6, 137.6, 134.5, 132.8, 130.8, 130.5, 129.9, 128.9, 128.3, 126.6, 124.14, 121.12, 21.8. HRMS (ESI-Q-TOF): *m*/*z* [M + H]^+^ calcd. for C_21_H_16_Br_1_N_3_O_2_S_1_: 454.02198; found: 454.0222.

*1-(4-Methoxyphenyl)-4-[(4-methylphenyl)sulfonyl]-5-phenyl-1H-1,2,3-triazole* (**4m**, known compound [22]). Prepared according to the general procedure using acetophenone 1a (60 mg, 0.50 mmol), sodium p-toluenesulfinate 2a (178 mg, 1.00 mmol), and 4-methoxyphenyl azide 3c (113 mg, 0.750 mmol). Reaction time was 24 h. Flash chromatography using EtOAc/petroleum ether 10–50% yielded 4m (130 mg, 0.312 mmol, 64%) as a white solid; mp 194–196 °C. ^1^H-NMR (400 MHz, CDCl_3_): δ (ppm) 7.82–7.73 (app. d, *J* = 8.3 Hz, 2H), 7.49–7.43 (m, 1H), 7.43–7.35 (m, 2H), 7.30–7.23 (m, 4H), 7.16–7.10 (app. d, *J* = 9 Hz, 2H), 6.86–6.80 (app. d, *J* = 9 Hz, 2H), 3.79 (s, 3H), 2.40 (s, 3H). ^13^C-NMR (101 MHz, CDCl_3_): δ (ppm) 160.6, 146.1, 144.9, 138.7, 137.9, 130.6, 130.5, 129.9, 128.7, 128.5, 128.3, 126.7, 124.7, 114.6, 55.7, 21.8. HRMS (ESI-Q-TOF): *m*/*z* [M + H]^+^ calcd. for C_22_H_19_N_3_O_3_S_1_: 454. 406.12198; found: 406.1220.

*1-Benzyl-4-[(4-methylphenyl)sulfonyl]-5-phenyl-1H-1,2,3-triazole* (**4o**), Prepared according to the general procedure using acetophenone **1a** (60 mg, 0.50 mmol), sodium p-toluenesulfinate **2a** (178 mg, 1.00 mmol), and benzyl azide **3d** (94 mL, 0.75 mmol). Reaction temperature was 35 °C and reaction time was 24 h. Flash chromatography using EtOAc/petroleum ether 10–40% yielded **4o** (35 mg, 0.090 mmol, 18%) as an off-white semi-solid. ^1^H-NMR (400 MHz, CDCl_3_): δ (ppm) 7.74–7.67 (app. d, *J* = 8.32, 2 H), 7.57–7.51 (m, 1H), 7.48–7.42 (m, 2H), 7.28–7.20 (m, 5H), 7.18–7.12 (m, 2H), 6.98–9.62 (m, 2H), 5.33 (s, 2H), 2.39 (s, 3H). ^13^C-NMR (101 MHz, CDCl_3_): δ (ppm) 146.2, 144.8, 139.0, 137.9, 134.0, 130.8, 130.2, 129.8, 129.0, 128.8, 128.8, 128.2, 127.9, 124.5, 52.7, 21.8. HRMS (ESI-Q-TOF): *m*/*z* [M + H]^+^ calcd. for C_22_H_19_N_3_O_2_S_1_: 390.12706; found: 390.1270.

### 3.3. Procedures for Mechanistic Studies

#### 3.3.1. Procedure A: Reaction under Argon Atmosphere

To a flame dried, Ar-flushed 10 mL Schlenck tube, sodium p-toluene sulfinate 2a (178 mg, 1.00 mmol, 2 eq.) and CuCl_2_ (6.7 mg, 10 mol%) were added. After evacuating and filling with argon three times, DMSO (dry, 2 mL), DBU (150 µL, 1.00 mmol, 2 eq.), TMEDA (15 µL, 0.05 mmol, 10 mol%) and phenyl azide 3a (83 μL, 0.75 mmol, 1.5 eq.) and acetophenone 1a (60 mg, 58 µL, 0.50 mmol) were added sequentially. After stirring for 24 h at 25 °C, 5 mL EtOAc and 3 mL NH_3_Cl were added and the mixture was transferred to a separation funnel along with 20 mL EtOAc. Then, 3 mL H_2_O was added in order to break the suspension. The organic phase was collected and following two more extractions (2 × 25 mL EtOAc), washed with brine, dried over Na_2_SO_4_, and concentrated in vacuo. TLC, ^1^H-NMR, and GC/MS showed only a trace of triazole 4a.

#### 3.3.2. Procedure B: Reaction in Presence of TEMPO

To a 10 mL round bottom flask, acetophenone **1a** (60 mg, 0.50 mmol), DMSO (dry, 2 mL), p-toluene sulfinate **2a** (178 mg, 1.00 mmol, 2 eq.), DBU (150 µL, 1.00 mmol, 2 eq.), TMEDA (15 µL, 0.05 mmol, 10 mol phenyl), azide **3a** (83 μL, 0.75 mmol, 1.5 eq.), and TEMPO (312 mg, 2 mmol, 4 eq.) were added sequentially. The reaction was initiated by addition of CuCl_2_ (6.7 mg, 10 mol%). After stirring the open vessel for 24 h at 25 °C, 5 mL EtOAc and 3 mL NH_3_Cl were added and the mixture was transferred to a separation funnel along with 20 mL EtOAc. Then, 3 mL H_2_O was added in order to break the suspension. The organic phase was collected and following two more extractions (2 × 25 mL EtOAc), washed with brine, dried over Na_2_SO_4_, and concentrated in vacuo. TLC, ^1^H-NMR, and GC/MS showed no triazole **4a** or sulfonyl ketone **5a**.

#### 3.3.3. Procedure C: Reaction of 1,1-Diphenylethylene (DPE)

To a 10 mL round bottom flask, acetophenone **1a** (60 mg, 0.50 mmol), DMSO (dry, 2 mL), p-toluene sulfinate **2a** (1.00 mmol, 2 eq.), DBU (150 µL, 1.00 mmol, 2 eq.), TMEDA (15 µL, 0.05 mmol, 10 mol%), phenyl azide **3a** (83 μL, 0.75 mmol, 1.5 eq.), and DPE (180 mg, 1.00 mmol, 2 eq.) were added sequentially. The reaction was initiated by addition of CuCl_2_ (6.7 mg, 10 mol%). After stirring the open vessel for 24 h at 25 °C, 5 mL EtOAc and 3 mL NH_3_Cl were added and the mixture was transferred to a separation funnel along with 20 mL EtOAc. Then, 3 mL H_2_O was added in order to break the suspension. The organic phase was collected and following two more extractions (2 × 25 mL EtOAc), washed with brine, dried over Na_2_SO_4_, and concentrated in vacuo. Flash chromatography with ethyl acetate/petroleum ether 10–40% as eluent afforded triazole **4a** (73 mg, 0.194 mmol, 39%) and radical trapping product **7** (14 mg, 0.0419 mmol, 4%). ^1^H-NMR for **7** was in accordance with the literature.

#### 3.3.4. Procedure D: Reaction of Phenacyl Chloride under Standard Conditions

To a 10 mL round bottom flask, phenacyl chloride **8** (77 mg, 0.50 mmol), DMSO (dry, 2 mL), p-toluene sulfinate **2a** (1.00 mmol, 2 eq.), DBU (150 µL, 1.00 mmol, 2 eq.), TMEDA (15 µL, 0.05 mmol, 10 mol%), and phenyl azide **3a** (83 μL, 0.75 mmol, 1.5 eq.) were added sequentially. The reaction was initiated by addition of CuCl_2_ (6.7 mg, 10 mol%). After stirring the open vessel for 24 h at 25 °C, 5 mL EtOAc and 3 mL NH_3_Cl were added and the mixture was transferred to a separation funnel along with 20 mL EtOAc. Then, 3 mL H_2_O was added in order to break the suspension. The organic phase was collected and following two more extractions (2 × 25 mL EtOAc), washed with brine, dried over Na_2_SO_4_, and concentrated in vacuo. Flash chromatography with ethyl acetate/petroleum ether 10–40% as eluent afforded triazole **4a** (19 mg, 0.05 mmol, 10%).

#### 3.3.5. Procedure E: Reaction of Phenacyl Chloride under Copper-Free Conditions

To a 10 mL round bottom flask, phenacyl chloride **8** (77 mg, 0.50 mmol), DMSO (dry, 2 mL), p-toluene sulfinate **2a** (1.00 mmol, 2 eq.), DBU (150 µL, 1.00 mmol, 2 eq.), TMEDA (15 µL, 0.05 mmol, 10 mol%), and phenyl azide **3a** (83 μL, 0.75 mmol, 1.5 eq.) were added sequentially. After stirring the open vessel for 24 h at 25 °C, 5 mL EtOAc and 3 mL NH_3_Cl were added and the mixture was transferred to a separation funnel along with 20 mL EtOAc. Then, 3 mL H_2_O was added in order to break the suspension. The organic phase was collected and following two more extractions (2 × 25 mL EtOAc), washed with brine, dried over Na_2_SO_4_, and concentrated in vacuo. Flash chromatography with ethyl acetate/petroleum ether 10–40% as eluent afforded triazole **4a** (75 mg, 0.20 mmol, 40%).

#### 3.3.6. Procedure F: Reaction of Acetophenone (**1a**) in Absence of Azide towards Ketosulfones **5a** and **5b**

To a 10 mL round bottom flask, acetophenone **1a**,**b** (0.50 mmol), DMSO (dry, 2 mL), sodium p-toluenesulfinate **2a** (178 mg, 1.00 mmol, 2eq), DBU (150 µL, 1.00 mmol, 2 eq.), and TMEDA (15 µL, 0.050 mmol, 10 mol%) were added sequentially. The reaction was initiated by addition of CuCl_2_ (6.7 mg, 10 mol%). After stirring the open vessel for 24 h at 25 °C, 5 mL EtOAc and 3 mL NH_3_Cl were added and the mixture was transferred to a separation funnel along with 20 mL EtOAc. Then, 3 mL H_2_O was added in order to break the suspension. The organic phase was collected and following two more extractions (2 × 25 mL EtOAc), washed with brine, dried over Na_2_SO_4_, and concentrated in vacuo. Flash chromatography with ethyl acetate/petroleum ether 10–30% as eluent afforded the title products as white solids.

*1-Phenyl-2-(toluene-4-sulfonyl)-ethanone* (**5a**, Known compound [74,75]). Prepared according to the general procedure using acetophenone **1a** (60 mg, 0.50 mmol). Reaction time was 24 h. Flash chromatography using EtOAc/petroleum ether 10–30% yielded **5a** (89 mg, 0.324 mmol, 65%) as a white solid; mp 105–106 °C (Lit. mp 105–106 °C) [75]. ^1^H-NMR (400 MHz, CDCl_3_): δ (ppm) 8.03–7.87 (app. d, *J* = 7.2 Hz, 2 H), 7.83–7.70 (app. d, *J* = 6.8 Hz, 2H), 7.67–7.56 (m, 1H), 7.54–7.42 (m, 2H), 7.38–7.26 (app. d, *J* = 6.8 Hz, 2H), 4.72 (s, 2H), 2.43 (s, 3H). ^13^C-NMR (101 MHz, CDCl_3_): δ (ppm) 188.3, 145.5, 135.88, 135.87, 134.4, 129.9, 129.4, 128.9, 128.7, 63.7, 21.8.

*1-(4-Trifluoromethylphenyl)-2-(toluene-4-sulfonyl)-ethanone* (**5b**, known compound [27]). Prepared according to the general procedure using 4′-(trifluoromethyl)acetophenone **1b** (94 mg, 0.50 mmol). Reaction time was 24 h. Flash chromatography using EtOAc/petroleum ether 10–30% yielded **5b** (113 mg, 0.330 mmol, 66%) as a white solid; mp 136–137 °C. ^1^H-NMR (400 MHz, CDCl_3_): δ (ppm) 8.14–8.00 (app. d, *J* = 8.2 Hz, 2H), 8.85–8.65 (m, 4H), 4.42–4.28 (app. d, *J* = 8.1 Hz), 4.76 (s, 2H), 2.44 (s, 3H). ^13^C-NMR (101 MHz, CDCl_3_): δ (ppm) 187.6, 145.8, 138.4, 135.6, 135.4 (q, *J* = 32.9 Hz), 130.0, 129.8, 128.6, 127.5, 125.9 (q, *J* = 3.7 Hz), 123.5 (q, *J* = 272.9 Hz), 119.4, 63.9, 21.8. ^19^F-NMR (377 MHz, CDCl_3_) δ (ppm) −63.29 (s, 1H). HRMS (ESI-Q-TOF): *m*/*z* [M + Na]^+^ calcd. for C_25_H_19_N_3_O_2_S_1_: 365.04299; found: 365.0427.

## 4. Conclusions

In conclusion, we have developed an efficient Cu-catalyzed three-component synthesis of 4-sulfonyl triazoles from aromatic ketones, azides, and sodium sulfinates, with air oxygen as terminal oxidant, operating at room temperature. This reaction involves a sequential oxidative sulfonylation of aromatic ketones/Dimroth cyclization. Preliminary mechanistic investigations indicate that both sulfonyl free radicals and organometallic Cu(III)-intermediates are involved.

## Figures and Tables

**Table 1:**
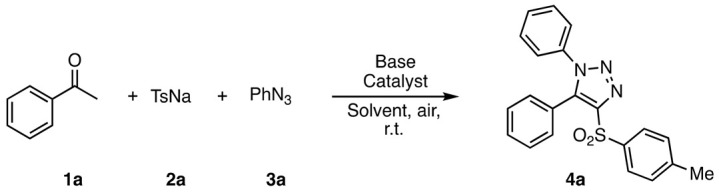
Optimization of reaction conditions ^a^.

Entry	Catalyst	Base	Base pKaH	Solvent	Ligand	Yield (%) ^b^
1	CuBr_2_	DBU	12	DMSO	none	56
2	CuCl_2_	DBU	12	DMSO	none	71 (72)
3	CuI	DBU	12	DMSO	none	47
4	Cu(OAc)_2_	DBU	12	DMSO	none	62
5	Cu(OTf)_2_	DBU	12	DMSO	none	64
6	CuCl_2_	Pyridine	3.4	DMSO	none	0
7	CuCl_2_	Et_3_N	9	DMSO	none	0
8	CuCl_2_	DBN	13.4	DMSO	none	52
9	CuCl_2_	K_2_CO_3_	10.3	DMSO	none	0
10	CuCl_2_	KOtBu	29.4	DMSO	none	0
11	CuCl_2_	DBU	12	DCM	none	0
12	CuCl_2_	DBU	12	EtOAc	none	5
13	CuCl_2_	DBU	12	ACN	none	7
14	CuCl_2_	DBU	12	DMF	none	24
15	CuCl_2_	DBU	12	EtOH	none	4
16	CuCl_2_	DBU	12	DMSO	TMEDA	81
17	CuCl_2_	DBU	12	DMSO	2,2’-bipyridine	67
18	CuCl_2_	DBU	12	DMSO	1,10-phenatroline	68
19	CuCl_2_	DBU	12	DMSO	Neocuproine	58
20	CuCl_2_	DBU	12	DMSO	TMEDA	84 ^d^
21	CuCl_2_	DBU	12	DMSO	TMEDA	77 ^e^
**22**	**CuCl_2_**	**DBU**	**12**	**DMSO**	**TMEDA**	**89 ^f^**
23	CuCl_2_	DBU	12	DMSO	TMEDA	50 ^g^
24	none	DBU	12	DMSO	TMEDA	Nr ^c^
25	CuCl_2_	DBU	12	DMSO	TMEDA	<5 ^h^

^a^ Reaction conditions: Acetophenone **1** (0.50 mmol), sodium p-toluene sulfinate **2** (1.00 mmol), DBU (1.00 mmol), catalyst (20%) in DMSO (3 mL) under air atmosphere. ^b^ Isolated yield. ^c^ n.r. = no reaction. ^d^ DMSO (2 mL). ^e^ DMSO (1 mL). ^f^ CuCl_2_/TMEDA (10 mol%), DMSO (2 mL). ^g^ CuCl_2_/TMEDA (5 mol%), DMSO (2 mL). ^h^ Reaction under argon atmosphere. The entry highlighted in bold corresponds to the optimized conditions.

## Data Availability

Data is contained within the article or supplementary material. FAIR Data is available as Supporting Information for Publication and includes the primary NMR FID files for compounds: **4a**, **4b**, **4c**, **4d**, **4e**, **4f**, **4g**, **4h**, **4i**, **4j**, **4k**, **4l**, **4m**, **4o**, **5a**, **5b**.

## Supplementary Materials

The following are available online. Table S1: Optimization of reaction conditions, copies and primary NMR FID files of the ^1^H, ^19^F, and ^13^C-NMR spectra.

## Sample Availability

Not available.
