# Supplementary material for: The Three-Component Synthesis of 4-Sulfonyl-1,2,3-triazoles via a Sequential Aerobic Copper-Catalyzed Sulfonylation and Dimroth Cyclization
Source: Molecules. 2021 Jan 22;26(3):581. doi: 10.3390/molecules26030581 (PMC7865689; doi:10.3390/molecules26030581)
Supplement: Supplementary file 1 [file molecules-26-00581-s001.zip › SI files-Final/MVH - SI - Molecules.docx]

**Supporting information**

**The three-component synthesis of 4-sulfonyl-1,2,3-triazoles via a sequential copper-catalyzed aerobic oxidative coupling and Dimroth cyclization**

Max Van Hoof, Santhini Pulikkal Veettil, Wim Dehaen*

Molecular Design and Synthesis, Department of Chemistry, KU Leuven, Celestijnenlaan 200F, B-3001 Leuven, Belgium

* Email: wim.dehaen@kuleuven.be

**Table of contents**

[Optimization table S2](#_Toc61779479)

[References S3](#_Toc61779480)

[NMR Spectra S4](#_Toc61779481)

# Optimization table

**Table S1 Optimization of reaction conditions^a^**

| \| **Entry** \| **Catalyst** \| **Base** \| **Base pKaH** \| **Solvent** \| **Ligand** \| **Reaction time** \| **Yield^b^** \| \| --- \| --- \| --- \| --- \| --- \| --- \| --- \| --- \| \| **(mol%)** \| **(equiv.)** \| **(mL)** \| **(mol%)** \| **(h)** \| **(%)** \| \| **1** \| CuBr_2_ (20) \| DBU (2) \| 12 \| DMSO (3) \| none \| 12 \| 56 \| \| **2** \| CuCl_2_ (20) \| DBU (2) \| 12 \| DMSO (3) \| none \| 12 \| 71 (72) \| \| **3** \| CuI (20) \| DBU (2) \| 12 \| DMSO (3) \| none \| 12 \| 47 \| \| **4** \| Cu(OAc)_2_ (20) \| DBU (2) \| 12 \| DMSO (3) \| none \| 12 \| 62 \| \| **5** \| Cu(OTf)_2_ (20) \| DBU (2) \| 12 \| DMSO (3) \| none \| 12 \| 64 \| \| **6** \| CuCl_2_ (20) \| Pyridine (2) \| 3.4 \| DMSO (3) \| none \| 12 \| 0 \| \| **7** \| CuCl_2_ (20) \| Et_3_N (2) \| 9 \| DMSO (3) \| none \| 12 \| 0 \| \| **8** \| CuCl_2_ (20) \| DBN (2) \| 13.4 \| DMSO (3) \| none \| 12 \| 52 \| \| **9** \| CuCl_2_ (20) \| K2CO3 (2) \| 10.3 \| DMSO (3) \| none \| 12 \| 0 \| \| **10** \| CuCl_2_ (20) \| KOtBu (2) \| 29.4 \| DMSO (3) \| none \| 12 \| 0 \| \| **11** \| CuCl_2_ (20) \| DBU (2) \| 12 \| DCM (3) \| none \| 12 \| 0 \| \| **12** \| CuCl_2_ (20) \| DBU (2) \| 12 \| EtOAc (3) \| none \| 12 \| 5 \| \| **13** \| CuCl_2_ (20) \| DBU (2) \| 12 \| ACN (3) \| none \| 12 \| 7 \| \| **14** \| CuCl_2_ (20) \| DBU (2) \| 12 \| DMF (3) \| none \| 12 \| 24 \| \| **15** \| CuCl_2_ (20) \| DBU (2) \| 12 \| EtOH (3) \| none \| 12 \| 4 \| \| **16** \| CuCl_2_ (20) \| DBU (1.5) \| 12 \| DMSO (3) \| none \| 12 \| 66 \| \| **17** \| CuCl_2_ (20) \| DBU (1.0) \| 12 \| DMSO (3) \| none \| 12 \| 60 \| \| **18** \| CuCl_2_ (20) \| DBU (0.6) \| 12 \| DMSO (3) \| none \| 12 \| 57 \| \| **19** \| CuCl_2_ (20) \| DBU (2) \| 12 \| DMSO (3) \| TMEDA \| 12 \| 81 \| \| **20** \| CuCl_2_ (20) \| DBU (2) \| 12 \| DMSO (3) \| 2,2'-bipyridine \| 24 \| 67 \| \| **21** \| CuCl_2_ (20) \| DBU (2) \| 12 \| DMSO (3) \| 1,10-phenatroline \| 24 \| 68 \| \| **22** \| CuCl_2_ (20) \| DBU (2) \| 12 \| DMSO (3) \| Neocuproine \| 24 \| 58 \| \| **23** \| CuCl_2_ (20) \| DBU (2) \| 12 \| DMSO (2) \| TMEDA \| 24 \| 84 \| \| **24** \| CuCl_2_ (20) \| DBU (2) \| 12 \| DMSO (1) \| TMEDA \| 24 \| 77 \| \| **25** \| **CuCl_2_ (10)** \| **DBU (2)** \| **12** \| **DMSO (2)** \| **TMEDA** \| **24** \| **89** \| \| **26** \| CuCl_2_ (5) \| DBU (2) \| 12 \| DMSO (2) \| TMEDA \| 24 \| 50 \| \| **27** \| none \| DBU (2) \| 12 \| DMSO (2) \| TMEDA \| 24 \| nr**^c^** \| \| **28** \| CuCl_2_ (10) \| DBU (2) \| 12 \| DMSO (2) \| TMEDA \| 24 \| < 5**^d^** \| |
| --- | --- | --- | --- | --- | --- | --- | --- | --- | --- | --- | --- | --- | --- | --- | --- | --- | --- | --- | --- | --- | --- | --- | --- | --- | --- | --- | --- | --- | --- | --- | --- | --- | --- | --- | --- | --- | --- | --- | --- | --- | --- | --- | --- | --- | --- | --- | --- | --- | --- | --- | --- | --- | --- | --- | --- | --- | --- | --- | --- | --- | --- | --- | --- | --- | --- | --- | --- | --- | --- | --- | --- | --- | --- | --- | --- | --- | --- | --- | --- | --- | --- | --- | --- | --- | --- | --- | --- | --- | --- | --- | --- | --- | --- | --- | --- | --- | --- | --- | --- | --- | --- | --- | --- | --- | --- | --- | --- | --- | --- | --- | --- | --- | --- | --- | --- | --- | --- | --- | --- | --- | --- | --- | --- | --- | --- | --- | --- | --- | --- | --- | --- | --- | --- | --- | --- | --- | --- | --- | --- | --- | --- | --- | --- | --- | --- | --- | --- | --- | --- | --- | --- | --- | --- | --- | --- | --- | --- | --- | --- | --- | --- | --- | --- | --- | --- | --- | --- | --- | --- | --- | --- | --- | --- | --- | --- | --- | --- | --- | --- | --- | --- | --- | --- | --- | --- | --- | --- | --- | --- | --- | --- | --- | --- | --- | --- | --- | --- | --- | --- | --- | --- | --- | --- | --- | --- | --- | --- | --- | --- | --- | --- | --- | --- | --- | --- | --- | --- | --- | --- | --- | --- | --- | --- | --- | --- | --- | --- | --- | --- | --- | --- | --- | --- | --- | --- | --- | --- | --- |

^a^ Reaction conditions: Acetophenone 1 (0.5 mmol), sodium p-toluenesulfinate 2 (1 mmol), DBU (1 mmol) under air atmosphere at room temperature. ^b^ Isolated yield. ^c^ n.r. = no reaction. ^d^ Reaction under argon atmosphere.

# References

Use the "Insert Citation" button to add citations to this document.

NMR Spectra

**1,5-Diphenyl-4-[(4-methylphenyl)sulfonyl]-1*H*-1,2,3-triazole (4a)**

^1^H NMR (400 MHz, CDCl_3_)

^13^C NMR (101 MHz, CDCl_3_)****

**1-Phenyl-4-[(4-methylphenyl)sulfonyl]-5-(4-trifluoromethylphenyl)-1*H*-1,2,3-triazole (4b)**

^1^H NMR (400 MHz, CDCl_3_)

^13^C NMR (101 MHz, CDCl_3_)****

^19^F NMR (377 MHz, CDCl_3_)

**1-Phenyl-4-[(4-methylphenyl)sulfonyl]-5-(4-fluorophenyl)-1*H*-1,2,3-triazole (4c)**

^1^H NMR (400 MHz, CDCl_3_)

^13^C NMR (101 MHz, CDCl_3_)

^19^F NMR (377 MHz, CDCl_3_)

**1-Phenyl-4-[(4-methylphenyl)sulfonyl]-5-(3-bromomethylphenyl)-1*H*-1,2,3-triazole (4d)**

^1^H NMR (400 MHz, CDCl_3_)

^13^C NMR (101 MHz, CDCl_3_)

**1-Phenyl-4-[(4-methylphenyl)sulfonyl]-5-(4-methylphenyl)-1*H*-1,2,3-triazole (4e)**

^1^H NMR (400 MHz, CDCl_3_)

^13^C NMR (101 MHz, CDCl_3_)

**1-Phenyl-4-[(4-methylphenyl)sulfonyl]-5-(4-methoxyphenyl)-1*H*-1,2,3-triazole (4f)**

^1^H NMR (400 MHz, CDCl_3_)

^13^C NMR (101 MHz, CDCl_3_)

**1-Phenyl-4-[(4-methylphenyl)sulfonyl]-5-(4-methoxyphenyl)-1*H*-1,2,3-triazole (4g)**

^1^H NMR (400 MHz, CDCl_3_)

^13^C NMR (101 MHz, CDCl_3_)

**1-Phenyl-4-[(4-methylphenyl)sulfonyl]-5-(1-naphthyl)-1*H*-1,2,3-triazole (4h)**

^1^H NMR (400 MHz, CDCl_3_)

^13^C NMR (101 MHz, CDCl_3_)

**1-Phenyl-4-[(4-methylphenyl)sulfonyl]-5-(2-naphtyl)-1*H*-1,2,3-triazole (4i)**

^1^H NMR (400 MHz, CDCl_3_)

^13^C NMR (101 MHz, CDCl_3_)

**1,5-Diphenyl-4-methylsulfonyl-1*H*-1,2,3-triazole (4j)**

^1^H NMR (400 MHz, CDCl_3_)

^13^C NMR (101 MHz, CDCl_3_)

**1,5-Diphenyl-4-[(4-chlorophenyl)sulfonyl]-1*H*-1,2,3-triazole (4k)**

^1^H NMR (400 MHz, CDCl_3_)

^13^C NMR (101 MHz, CDCl_3_)

**1-(4-Bromophenyl)-4-[(4-methylphenyl)sulfonyl]-5-phenyl-1*H*-1,2,3-triazole (4l)**

^1^H NMR (400 MHz, CDCl_3_)

^13^C NMR (101 MHz, CDCl_3_)

**1-(4-Methoxyphenyl)-4-[(4-methylphenyl)sulfonyl]-5-phenyl-1*H*-1,2,3-triazole (4m)**

^1^H NMR (400 MHz, CDCl_3_)

^13^C NMR (101 MHz, CDCl_3_)****

**1-Benzyl-4-[(4-methylphenyl)sulfonyl]-5-phenyl-1*H*-1,2,3-triazole (4o)**

^1^H NMR (400 MHz, CDCl_3_)

^13^C NMR (101 MHz, CDCl_3_)

**1-Phenyl-2-(toluene-4-sulfonyl)-ethanone (5a)**

^1^H NMR (400 MHz, CDCl_3_)

^13^C NMR (101 MHz, CDCl_3_)

**1-(4-Trifluoromethylphenyl)-2-(toluene-4-sulfonyl)-ethanone (5a)**

^1^H NMR (400 MHz, CDCl_3_)

^13^C NMR (101 MHz, CDCl_3_)

^19^F NMR (377 MHz, CDCl_3_)
